# High prevalence of reverse transcriptase inhibitors associated resistance mutations among people living with HIV on dolutegravir-based antiretroviral therapy in Francistown, Botswana

**DOI:** 10.1093/jac/dkae472

**Published:** 2025-01-07

**Authors:** Ontlametse T Choga, Goitseone M Lemogang, Wonderful T Choga, Gaonyadiwe Muzanywa, Thembinkosi M Shadreck, Charity Ralegoreng, Dorcas Maruapula, Natasha O Moraka, Catherine K Koofhethile, Patrick T Mokgethi, Kedumetse Seru, Boitumelo J L Zuze, Patience Montshosi, Irene Gobe, Modisa S Motswaledi, Rosemary Musonda, Mpaphi B Mbulawa, Joseph Makhema, Roger Shapiro, Shahin Lockman, Tony Chebani, Judith Nawa, Lindani Bochena, Sikhulile Moyo, Simani Gaseitsiwe

**Affiliations:** Research Laboratory, Botswana Harvard Health Partnership, Gaborone, Botswana; Department of Medical Sciences, Faculty of Allied Health Professions, University of Botswana, Gaborone, Botswana; Research Laboratory, Botswana Harvard Health Partnership, Gaborone, Botswana; Research Laboratory, Botswana Harvard Health Partnership, Gaborone, Botswana; Department of Medical Sciences, Faculty of Allied Health Professions, University of Botswana, Gaborone, Botswana; Research Laboratory, Botswana Harvard Health Partnership, Gaborone, Botswana; Research Laboratory, Botswana Harvard Health Partnership, Gaborone, Botswana; Research Laboratory, Botswana Harvard Health Partnership, Gaborone, Botswana; Research Laboratory, Botswana Harvard Health Partnership, Gaborone, Botswana; Research Laboratory, Botswana Harvard Health Partnership, Gaborone, Botswana; Department of Medical Sciences, Faculty of Allied Health Professions, University of Botswana, Gaborone, Botswana; Research Laboratory, Botswana Harvard Health Partnership, Gaborone, Botswana; Department of Immunology and Infectious Diseases, Harvard T.H. Chan School of Public Health, Boston, MA, USA; Research Laboratory, Botswana Harvard Health Partnership, Gaborone, Botswana; Department of Biological Sciences, Faculty of Science, University of Botswana, Gaborone, Botswana; Research Laboratory, Botswana Harvard Health Partnership, Gaborone, Botswana; Research Laboratory, Botswana Harvard Health Partnership, Gaborone, Botswana; Department of Medical Sciences, Faculty of Allied Health Professions, University of Botswana, Gaborone, Botswana; Research Laboratory, Botswana Harvard Health Partnership, Gaborone, Botswana; Department of Medical Sciences, Faculty of Allied Health Professions, University of Botswana, Gaborone, Botswana; Department of Medical Sciences, Faculty of Allied Health Professions, University of Botswana, Gaborone, Botswana; Research Laboratory, Botswana Harvard Health Partnership, Gaborone, Botswana; Data Warehouse, Botswana Ministry of Health, Gaborone, Botswana; Research Laboratory, Botswana Harvard Health Partnership, Gaborone, Botswana; Department of Immunology and Infectious Diseases, Harvard T.H. Chan School of Public Health, Boston, MA, USA; Research Laboratory, Botswana Harvard Health Partnership, Gaborone, Botswana; Department of Immunology and Infectious Diseases, Harvard T.H. Chan School of Public Health, Boston, MA, USA; Research Laboratory, Botswana Harvard Health Partnership, Gaborone, Botswana; Department of Immunology and Infectious Diseases, Harvard T.H. Chan School of Public Health, Boston, MA, USA; Division of Infectious Diseases, Brigham & Women’s Hospital, Boston, MA, USA; Data Warehouse, Botswana Ministry of Health, Gaborone, Botswana; Data Warehouse, Botswana Ministry of Health, Gaborone, Botswana; Nyangabgwe HIV Reference Laboratory, Botswana Ministry of Health, Francistown, Botswana; Research Laboratory, Botswana Harvard Health Partnership, Gaborone, Botswana; Department of Immunology and Infectious Diseases, Harvard T.H. Chan School of Public Health, Boston, MA, USA; School of Health Systems and Public Health, Faculty of Health Sciences, University of Pretoria, Pretoria, South Africa; Department of Pathology, Division of Medical Virology, Stellenbosch University, Cape Town, South Africa; Research Laboratory, Botswana Harvard Health Partnership, Gaborone, Botswana; Department of Immunology and Infectious Diseases, Harvard T.H. Chan School of Public Health, Boston, MA, USA

## Abstract

**Objectives:**

We assessed HIV-1 drug resistance profiles among people living with HIV (PLWH) with detectable viral load (VL) and on dolutegravir-based antiretroviral therapy (ART) in Botswana.

**Methods:**

The study utilised available 100 residual HIV-1 VL samples from unique PLWH in Francistown who had viraemia at-least 6 months after initiating ART in Botswana’s national ART program from November 2023 to January 2024. Viraemia was categorized as low-level viraemia (LLV) (VL: 200–999 copies/mL) or virologic failure (VF) (VL ≥1000 copies/mL). HIV-1 protease, reverse transcriptase and integrase genes were sequenced using an in-house next-generation sequencing Oxford nanopore technology. HIV-1 drug resistance mutations (DRMs) were identified using the HIVdb Program in the Stanford HIV drug resistance database and compared between VL groups.

**Results:**

Among 100 participants, 83.0% were on dolutegravir-based, 10.0% were on non-dolutegravir-based ART and 7.0% had unknown/undocumented ART regimens. Thirty (30%) participants had LLV and 70 (70%) had VF. Among 58 successfully sequenced, 32.8% [95% Confidence Interval (CI): 21.8–46.0] had DRMs to any drug class, 33.3% (4/12) in the LLV group and 32.6% (15/46) in the VF group. Among individuals on dolutegravir-based ART, the overall HIV DRMs were 34.8% (95% CI: 22.7–49.2). By VL groups, 40.0% (95% CI: 16.8–68.7) and 33.3% (95% CI: 20.2–50.0) had DRMs at LLV and VF, respectively.

**Conclusions:**

A high but similar prevalence of any DRMs was observed among individuals with LLV and those with VF on dolutegravir-based therapy. Monitoring DRMs in individuals with detectable VL is crucial for preserving dolutegravir-based ART.

## Introduction

Botswana implemented the use of dolutegravir in 2016 as part of the preferred first-line and second-line combination antiretroviral therapy (ART).^1^ Dolutegravir is a second-generation INSTI with high efficacy, tolerability, limited drug–drug interactions and a high barrier to resistance.^2,3^ Despite major advances in the development of antiretroviral (ARV) drugs and ART treatment guidelines,^4,5^ Botswana, like other middle-income countries, continues to face challenges such as access to adherence support, viral load (VL) and resistance testing, recycling of drugs, limited HIV care specialists, drug stock-outs and lack of ancillary healthcare services.^6,7^ These challenges may facilitate the emergence of drug-resistant HIV-1 variants amid the widespread use of dolutegravir-based ART. The presence of HIV drug resistance mutations (DRMs) among people living with HIV (PLWH) on dolutegravir is associated with non-suppression.^8–11^ Therefore, HIV drug resistance monitoring among PLWH with detectable VL in the era of dolutegravir-based ART is a necessity to preserve future ART options. To date, no study has characterized HIV-1 DRMs in PLWH on dolutegravir-based ART in the Botswana National ART program. This study characterized HIV DRMs among PLWH with detectable VL > 200 copies/mL who were predominantly on dolutegravir-based ART in the central HIV VL testing laboratory in Francistown, Botswana.

## Methods

### Study population, size and design

A cross-sectional study was conducted using residual HIV VL plasma samples of PLWH enrolled in the Botswana National ART program receiving care in Francistown and surrounding healthcare facilities. National Program HIV-1 RNA testing labs use Abbott Realtime HIV-1 assay on the automated m2000rt/m2000sp system (Abbott Laboratories, Wiesbaden, Germany) with a lower limit of detection of 40 copies/mL. Amongst PLWH whose VL tests were conducted between November 2023 and January 2024 at Nyangabgwe HIV Reference Laboratory, all available samples with a detectable VL > 200 copies/mL and a minimum of 150 µL were selected for HIV drug resistance testing. Individuals who were on ART for at least 6 months were included. Demographics (age, gender), ART initiation date, ART regimen history and retrospective longitudinal HIV-1 VL measurements were extracted from medical records. For individuals without ART initiation date, the baseline CD4+T cell count test date was used as the date of initiation and where baseline CD4+T cell count test date was not available, the first VL test date recorded was used and 90 days were subtracted to impute the ART initiation date as VL testing is firstly performed at 3 months post ART initiation, according to Botswana HIV treatment guidelines, followed by 6-monthly VL measurements.^12^

### Ethical consideration

The Botswana Health Research and Development Committee (HRDC) provided ethics approval with a waiver of informed consent. Clinical data were extracted by the Ministry of Health and assigned a unique Study number.

### HIV genotyping

HIV RNA was extracted from 140 μL of plasma using the QIAamp viral RNA mini kit (QIAGEN, Hilden, Germany). Complementary DNA strand from protease (PR), reverse transcriptase (RT) of HIV-1 pol was amplified as previously described^13^ while integrase (IN) of HIV-1 pol was amplified using a protocol previously described.^14^

The library preparation protocol follows the PCR tiling of the SARS-CoV-2 virus with rapid barcoding and the Midnight RT PCR Expansion (SQK-RBK110.96 and EXP-MRT001), PCR Version: MRT_9127_v110_revH_14Jul2021 (https://nanoporetech.com/document/pcr-tiling-of-sars-cov-2-virus-with-rapid-barcoding-and-midnight). The amplified amplicons were quantified with a Nanodrop Spectrophotometer 1000 prior to rapid barcoding. After barcoding, all amplicons were pooled and purified using SPRI beads. The concentration and purity of the pooled product were then measured using the Qubit^™^ 4.0 Fluorometer with the Qubit dsDNA HS Assay Kit. The prepared DNA library was loaded into the SpotON port of the flow cell and sequenced on the Oxford Nanopore Technology (ONT) GridION.

### HIV drug resistance analysis

The unprocessed paired-end or single sequence reads obtained from ONT were analysed using Genome Detective (https://www.genomedetective.com). Genome Detective was used for quality control, removing adapters, filtering low-quality sequence reads, performing reference-based assembly to HXB2 and generating consensus sequences in FASTA format. The Fasta files generated were uploaded in the Stanford HIV drug resistance database HIVdb Program (https://hivdb.stanford.edu/hivdb/by-patterns/) to identify known HIV-1 DRMs associated with PI, NNRTI, NRTI and INSTI at ≥20% allele frequency. The level of antiretroviral drug (ARV) resistance was predicted according to the Stanford HIV DRM penalty scores and resistance interpretation. Individuals with intermediate-level and high-level resistance were considered to have drug resistance.

### Statistical analysis

Categorical variables were compared between VL groups using a chi-square test. Continuous variables were compared using the Wilcoxon rank sum test. Proportions of individuals with at least one HIV drug resistance mutation in the LLV and VF groups were compared using a comparison of proportions test. Confidence intervals for proportions were estimated using the Wilson Score Method applying the population sample weights. *P*-values ≤0.05 were considered statistically significant. Data analysis was performed using STATA version 16.

## Results

### Baseline demographics

**Figure 1.**
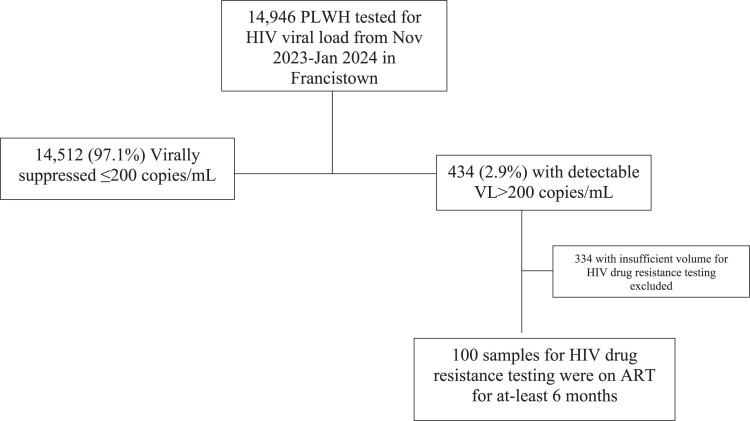
Selection of study participants.

Samples from 14 946 unique PLWH were received for VL testing from November 2023 to January 2024; HIV-1 RNA was undetectable in 97.1% (14 512). Among 434 participants with detectable VL, and on ART for at least 6 months in this period and have sufficient volume for testing were utilized in this study (Figure 1). Of these, a total of 30.0% (30) had LLV while 70.0% (70) had VF. When stratifying LLV by LLV ranges, 17 had medium LLV which is VL: 200–400 copies/mL while 13 had high-LLV (VL:401–999 copies/mL). Fifty-nine percent of the 100 individuals were females with a median age of 39 years (Q1, Q3: 29.5, 47) years. Individuals with LLV were significantly older than individuals with VF (*P* = 0.05) (Table 1). Current ART regimen data were available for 93 PLWH, of whom 89.2% (83) were on dolutegravir-based ART and 10.8% (10) were on NNRTI-based ART (*P* = 0.03). Out of the 83 individuals currently on dolutegravir-based ART, 38.6% (32) were initiated on dolutegravir as their first-line treatment, while 61.4% (51) transitioned from other ART regimens to dolutegravir following Botswana guideline updates, rather than due to VF. Amongst these 51, 14 were on efavirenz (EFV) or nevirapine (NVP)-based ART while 37 were initiated on ART prior to dolutegravir-based regimens, however, their specific ART options were not documented.

**Table 1.** Baseline demographics and HIV drug resistance profiles stratified by viral load groups

Variables	Total *N* = 100 (%)	LLV (VL: 200–1000 copies/mL) *N* = 30 (%)	VF (VL ≥ 1000 copies/mL) *N* = 70 (%)	*P*-values
Gender				0.31
Male	41 (41.0)	10 (33.3)	31 (44.3)	
Females	59 (59.0)	20(67.7)	39 (55.7)	
Median Age in years (Q1, Q3)	39 (29.5, 47)	43.0 (33, 52)	38, 43)	0.05^a^
ART initiation years				0.07
Prior June 2016	68 (68.0)	25 (83.3)	43 (61.4)	
Post June 2016	32 (32.0)	5 (16.7)	27(38.6)	
Historical ART regimen	*N* = 40	*N* = 13	*N* = 27	0.3
Dolutegravir-based ART	20 (50.0)	9 (69.2)	11 (40.7)	
Non-dolutegravir-based ART	20 (50.0)	4 (30.8)	16 (59.3)	
Current ART regimen	*N* = 93	*N* = 30	*N* = 63	0.03
Dolutegravir-based ART	83 (89.2)	27 (90.0)	56 (88.9)	
Non-dolutegravir-based ART	10 (10.8)	3 (10.0)	7 (11.1)	
Botswana Citizen				0.35
Yes	93 (93.0)	29 (96.7)	64 (91.4)	
No	7 (7.0)	1 (3.3)	6 (8.6)	
VL episode				0.1
Single	76 (76.0)	20 (66.7)	56 (80.0)	
Confirmed	20 (20.0)	7 (23.3)	13 (18.6)	
Persistent	4 (4.0)	3 (10)	1 (1.4)	
	*N* = 58	*N* = 12	*N* = 46	0.99
Overall HIV DRMS [95% CI:]	19 (32.8%) [95% CI: 21.8–46.0]	4(33.3%) [95% CI: 13.8–60.9]	15 (32.6%) [95% CI: 20.9–47.0]	
HIV DR classes				
PI (*n* = 49)	3 (6.1%) [95% CI: 2.1–16.5]	0 (0.0%)	3 (7.5%) [95% CI:2.6–19.9]	N/A
NRTI (*n* = 49)	6(12.2%) [95% CI: 5.7–24.2]	1 (11.1%) [95% CI:2.0–43.5]	5 (12.5%) [95% CI:5.5–26.1]	0.90
NNRTI (*n* = 49)	13(26.5) [95% CI: 16.2–40.3]	3 (33.3%) [95% CI:12.1–64.5]	10 (25.0%) [95% CI:14.2–40.2]	0.62
INSTI (*n* = 51)	3(5.9%) [95% CI:2.0–15.9]	1(9.1%) [95% CI:1.6–37.7]	2 (5.0%) [95% CI:1.4–16.5]	0.56

ART, antiretroviral therapy; dolutegravir, based ART-other regimens with dolutegravir; LLV, low-level viraemia; LPV, based ART-other regimens with lopinavir; N, number of people living with HIV; *n*, Total number of sequences available for each HIV drug class; Non-dolutegravir, other regimens with nevirapine or efavirenz; VF, virologic failure; 95% CI, 95% Confidence Intervals; Single, a single instance of LLV or VF; Confirmed, two consecutive LLV or VF measurements 6 months apart; Persistent, at-least three consecutive LLV or VF measurements within 12 ± 2 months. The prevalence of PI, NRTI and NNRTI was calculated using a denominator of 9 of PLWH with PRRT sequences in LLV while 40 was used in the VF group. Here 11 denominator was used for INSTI-associated resistance mutations in LLV while 40 was used in VF.

^a^ *P*-value calculated with Wilcoxon rank sum test.

### Amplification and genotyping outcomes by VL groups

A total of 68.0% (68/100) of samples were successfully amplified for HIV PRRT regions, by VL groups; 53.3% (16/30) were amplified at LLV compared with 74.3% (52/70) at VL ≥ 1000 copies/mL (*P*-value = 0.05). For the HIV integrase region, 71.0% of the samples were successfully amplified. The sequencing success was 72.1% (49/68) and 71.8% (51/71) for HIV PRRT and integrase regions, respectively. Higher HIV integrase sequencing success was achieved in samples with VL ≥ 1000 copies/mL at 76.9% (40/52) compared with samples with LLV at 52.4% (11/21) (*P*-value = 0.02). From 68 HIV PRRT-generated amplicons, a total of 56.3% (9/16) and 78.4% (40/51) were successfully sequenced at LLV and VF, respectively (Table S1, available as Supplementary data at *JAC* Online).

### Overall prevalence of HIV DRMs stratified by VL groups

Among 58 generated sequences, 32.8% [95% Confidence Interval (CI): 21.8–46.0] had at least one HIV DRM. PLWH with LLV (33.3%) had the same prevalence of HIV DRMs as those with VL ≥ 1000 copies/mL (32.6%) (*P*-value = 0.99) shown in Table 1. Of these PLWH with DRMs, 37.0% (17/46) had DRMs at a single instance of LLV or VF, 22.2% (2/9) in the confirmed VL group (two consecutive LLV or VF measurements) and none at persistent (at-least three consecutive) LLV or VF measurements. Table 2 shows the description of 19 PLWH with HIV DRMs and their resistance levels towards different ARV drugs.

**Table 2.** Characteristics of PLWH with HIV DRMs

Characteristics	ART initiation date	Previous ART regimen	Current ART regimen	Previous VL results (copies/mL)	HIV DRMs	ARV resistance levels
Age range: 20–30 Sex: Male Citizen: Yes	2003	ND	TLD	1300, 430, 3200, 4575, 8900, 3000, 5500, 5700, 5700, 13 000, 1900, <400*(6), 30, 30, 2406	INSTI: N155H, Q95K NRTI: M184V, T215Y NNRTI: K103N, V108I PI: None	Intermediate: ABC, AZT, D4T, DDI and CAB. High-level: FTC, 3TC, EFV, NVP, EVG and RAL.
Age range: 40–50 Sex: Male Citizen: No	2023	ND	TLD	1326	INSTI: R263K, E157Q NRTI: M41L, E44A, D67N, M184V, L210W, T215Y, K219E NNRTI: K103N, E138G, Y181C PI: None	Intermediate: ETR, DTG, BIC, EVG and RAL. High-level: ABC, AZT, D4T, DDI, FTC, TDF, EFV, NVP, RPV, CAB.
Age range: 50–60 Sex: Female Citizen: Yes	2008	AZT/3TC/EFV	TLD	<400*(7), 30, 30, 0, 30, 30, 30, 790	INSTI: G118R, E138K, R263K, L94M NRTI: None NNRTI: None PI: None	Intermediate: None High-level: BIC, CAB, DTG, EVG, RAL
Age range: 30–40 Sex; Female Citizen: Yes	2008	ND	TLD	499, <400, 55, 2989	INSTI: None NNRTI: None NNRTI: K103N PI: None	Intermediate: None High-level: EFV, NVP
Age range: 60–70 Sex: Male Citizen: Yes	2015	ND	TLD	4000, 43 507, 22 350, 1290, 76 091, 643, 3475, 500, 211, 1452, 131, 1452	INSTI: FS NRTI: D67N, T69D, L74I, Y115F, M184V, K219Q NNRTI: None PI: V82L, L90M, L33F, Q58E, G73T	Intermediate: LPVr, AZT, D4T, TDF High-level: ATVr, FPVr, DRVr, IDVr, NFV, SQVr, TPVr, ABC, DDI, FTC, 3TC
Age range: 60–70 Sex: Male Citizen: Yes	2016	ND	TLD	<400*(7), 30, 85 067, 30, 383, 1028, 685, 30, 30, 59 343	INSTI: None NRTI: None NNRTI: None PI: Q58E	Intermediate: TPVr High-level: None
Age range: 30–40 Sex: Male Citizen: Yes	2014	TDF/FTC/EFV	TLD	<400, <400, <400, 1118, <400, <400, 30, 89 154	INSTI: None NRTI: None NNRTI: E138A PI: None	Intermediate: RPV High-level: None
Age range: 20–30 Sex: Male Citizen: Yes	2016	ND	ND	<400*(5), 30, 45 697	INSTI: FS NRTI: None NNRTI: E138A PI: None	Intermediate: RPV High-level: None
Age range: 50–60 Sex: Male Citizen: Yes	2014	3TC	TLD	<400*(7), 40 082	INSTI: None NRTI: M184V NNRTI: None PI: None	Intermediate: ABC High-Level: FTC, 3TC
Age range: 20–30 Sex: Female Citizen: Yes	2015	ND	TLD	<400*(7), 627, <400*(5), 318	INSTI: None NRTI: None NNRTI: E138A PI: None	Intermediate: RPV High-level: None
Age range: 40–50 Sex: Female Citizen: Yes	2008	ND	TLD	59 000, 11 000, <400, <400*(13), 964 822, 20 134, 30, 30, 30, 30, 30, 1 953 806, 476	INSTI: None NRTI: A62V, K65R, S68G NNRTI: K101E, Y181C, G190A PI: None	Intermediate: FTC, 3TC, DOR High-level: D4T, DDI, TDF, EFV, ETR, NVP, RPV
Age range: 20–30 Sex: Female Citizen: No	2022	ND	TLD	7292, 181, 3523	INSTI: FS NRTI: None NNRTI: None PI: V82L	Intermediate: FPVr, TPVr High-level: None
Age range: 10–20 Sex: Female Citizen: Yes	2022	AZT/3TC/NVP	AZT/3TC/NVP	543, <400(35), 8448, 94, 30, 30, 1525, 311, 30, 30, 33 190	INSTI: None NRTI: None NNRTI: E138A PI: None	Intermediate: RPV High-level: None
Age range: 30–40 Sex: Female Citizen: Yes	2016	TDF/FTC/EFV	TLD	<400*(10), 997, 30, 20, 57,1850	INSTI: None NRTI: None NNRTI: V108I PI: None	Intermediate: DOR High-level: EFV, NVP
Age range: 40–50 Sex: Male Citizen: Yes	2011	TDF/FTC/NVP	TDF/FTC/NVP	<400*(10), 20, 30, 30, 7 500 000	INSTI: None NRTI: None NNRTI: None PI: V82L	Intermediate: TPVr High-level: None
Age range: 20–30 Sex: Female Citizen: Yes	2023	ND	TLD	133 674, 1574	INSTI: None NRTI: M184I NNRTI: V108I, Y181V, Y188F, G190S PI: None	Intermediate: ABC High-level: FTC, 3TC, DOR, EFV, ETR, RPV
Age range: 40–50 Sex: Female Citizen: Yes	2007	ND	TLD	670 000, <400*(8), 1000, 670, 750 000, 417 512, 367 265, 166 121, 16 000, 280 000, 470 000, <400, 29 643, 2994, 3384, <400, 2905, 991, 1497, <400, 2840, 1737, 1268, 2060, 6017, 7139, 30, 126 440, 76 054, 172 859, 145 299	INSTI: None NRTI: None NNRTI: V179D PI: None	Intermediate: None High-level: None
Age range: 20–30 Sex: Female Citizen: Yes	2016	ND	TLD	5300, 28 575, 15 000, 11 000, <400, <400, <400, 19 000, 10 240, 13 541, <400, 5518, 38 247, 50 084, 56 940, 30, 30, 44 530, 1527, 44 339	INSTI: None NRTI: None NNRTI: K101P, K103N PI: None	Intermediate: None High-level: EFV, ETR, NVP, RPV
Age range: 40–50 Sex: Male Citizen: Yes	2009	ND	TLD	<400(13), 30, 30, 30, 206	INSTI: FS NRTI: None NNRTI: E138A PI: None	Intermediate: RPV High-level: None

3TC, lamivudine; ABC, abacavir; ATV/r, atazanavir/ritonavir; AZT, zidovudine; BIC, bictegravir; CAB, cabotegravir; DDI, didanosine; DOR, doravirine; DTG, dolutegravir; DRMs, drug resistance mutations; DTG, dolutegravir; D4T, stavudine; EFV, efavirenz; ETR, etravirine; EVG, elvitegravir; FTC, emtricitabine; FPV/r, fosamprenvir/ritonavir; FS, failed sequencing; FVL, first VL measurement was genotyped; IDV/r, indinavir/ritonavir; INSTI, integrase strand transfer inhibitors; LLV, low-level viraemia; LPV/r, lopinavir/ritonavir; N, number of sequences available; ND, not documented; NFV, nelfinavir; NNRTI, non-nucleoside reverse transcriptase inhibitors; NRTI, nucleoside reverse transcriptase inhibitors; NVP, nevirapine; RAL, raltegravir; RPV, rilpvirine; TLD, tenofovir disoproxil fumarate/lamivudine/dolutegravir; TDF, tenofovir disoproxil fumarate; TPV, tipranavir.

*The consecutive VL measurement of the same value.

()The times that the same VL measurement value was recorded.

**Figure 2.**
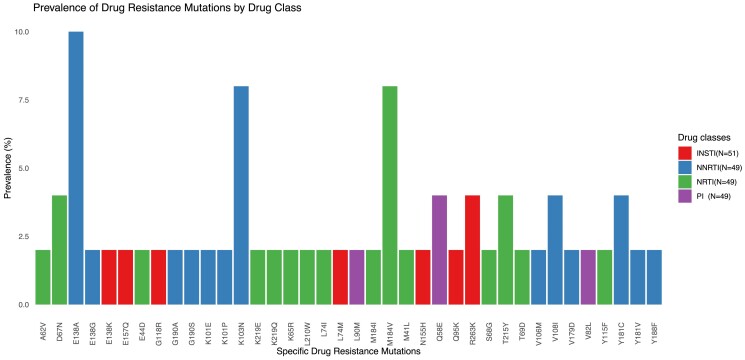
HIV DRMs by drug classes. INSTI, Integrase strand transfer inhibitors; NNRTI, Non-nucleoside reverse transcriptase inhibitors; NRTI, Nucleoside reverse transcriptase inhibitors; PI, Protease inhibitors.

The most 3 predominant DRMs with a prevalence >5% were E138A (10.2%), K103N (8.2%) and M184V (8.2%) (Figure 2). NNRTI-associated resistance mutations were most common (in 26.5% of PLWH) with DRM related to rilpivirine (RPV) in 18.0%, to EFV in 14.0% and to NVP in 14.0%. Three (5.2%) PLWH had INSTI-associated resistance mutations, one with potential low-level dolutegravir-associated resistance (N155H and Q95K mutations), one intermediate (R263K and E157Q mutations) and the remaining with high-level dolutegravir-associated resistance (G118R, E138K, R263K and L94M mutations).

### HIV drug resistance among PLWH on dolutegravir-based ART

Amongst 58 PLWH with HIV-1 sequences, 53 had current ART regimen data. Of these, 86.8% (46) were on dolutegravir-based ART. Among 46 individuals on dolutegravir-based ART, 21.7% (10) had LLV while 78.3% (36) had VF. A total of 32 were initiated on non-dolutegravir ART from 2003 to 2016 (and later switched to dolutegravir following Botswana guideline updates, rather than due to VF) while 14 were initiated from 2017 to 2023 (during the era of dolutegravir-based ART). Among 46 PLWH on dolutegravir-based ART, 34.8% (16) had at least one DRM; 40.0% (4/10) at LLV and 33.3% (12/36) at VF had at least one DRM. Of these 16, 13 were initiated from 2003 to 2016 and 3 from 2022 to 2023. Amongst PLWH on dolutegravir-based ART, 27.5% (11) had NNRTI resistance, 15.0% (6) had NRTI resistance, 5.0% (2) had PI resistance and 7.3% (3) had INSTI-associated resistance. Among 16 with DRMs, only two of these participants had HIV DRMs on dolutegravir first-line based ART while 14 were initially on other ART options before dolutegravir-based ART (three were on EFV-based ART, one on lamivudine while 10 their initial ART information was not documented).

## Discussion

We conducted a cross-sectional study to characterize HIV DRMs associated with each class of antiretrovirals in a small cohort of PLWH enrolled in the Botswana National ART program and experiencing detectable VL >200 copies/mL on ART. We report a prevalence of 34.8% of having at least one HIV DRM (conferring low-level or greater drug resistance) among PLWH on dolutegravir-based ART who had detectable VL, without differences by LLV or VF. We identified a low prevalence of dolutegravir-resistance-associated mutations (5.0%), but persistent resistance mutations associated with NNRTIs despite limited use in current ART regimens.

The reported prevalence of HIV DRMs in this cohort was similar to that of 32% from the previous study of PLWH on deep salvage dolutegravir therapy in Botswana,^15^ which indicated that ART post-exposure before dolutegravir may increase the risk of HIV DRMs. Our observed low prevalence of dolutegravir-associated resistance among PLWH with viraemia on dolutegravir-based ART aligns with previous studies that have indicated rare dolutegravir-associated resistance among PLWH who are failing dolutegravir-based first-line ART.^16–18^ A previous study did not detect dolutegravir-associated resistance among virologically failing PLWH who initiated dolutegravir as the first-line ART, though it reported 12.7% with INSTI-associated resistance.^19^ Although we do not have information on how many PLWH on dolutegravir in this study had previous ART regimens, we believe that 87.5% (14/16) of those with DRMs on dolutegravir-based ART were exposed to other ART options, as their ART initiation date was before the introduction of dolutegravir in Botswana. This study identified three individuals with INSTI resistance, two of whom were on dolutegravir-based second-line ART, suggesting that prior ART exposure may contribute to dolutegravir resistance. In addition, we observed a unique case of an individual with dolutegravir resistance on dolutegravir-based first-line ART. However, it is possible that this individual was infected with a resistant strain. Individuals may be experiencing treatment failure because they were switched to dolutegravir while their VL was suppressed, with DRMs primarily driven by an unoptimized background regimen rather than dolutegravir itself. Factors associated with the emergence of dolutegravir-associated resistance include poor treatment adherence, drug interactions and viral factors such as a high baseline VL.^20–22^ These findings emphasize the importance of monitoring resistance patterns to optimize ART strategies.

Some of the study participants had INSTI-associated resistance mutations including G118K, E138K, R263K, N188H, Q97K, L94M and E157Q which may impact the use of dolutegravir. G118R mutation is associated with high-level resistance to all INSTI-based ART^16^ except bictegravir,^23^ this mutation is usually reported among PLWH who are virologically failing and have emergent HIV drug resistance on dolutegravir. This mutation was reported in this study among two individuals whereby one participant has a VL of 1326 copies/mL with 12 HIV DRMs and the other has a VL of 790 copies/mL with 4 HIV DRMs. Mutation N155H alone is associated with high-level resistance towards raltegravir and elvitegravir^24^ but low-level resistance towards dolutegravir, bictegravir and cabotegravir^25^ among PLWH on raltegravir, elvitegravir, dolutegravir and cabotegravir.^18^ Our study reports one participant with mutation N155H with accessory mutation Q97K and this individual was susceptible to dolutegravir and bictegravir. E157Q is a naturally occurring polymorphism that confers some resistance to raltegravir and elvitegravir.^26^ However, it may affect the emergence of R263K and resistance to dolutegravir.^27,28^ A combination of these two mutations was reported in an individual with intermediate resistance toward raltegravir, elvitegravir, dolutegravir and bictegravir while high-level resistance was detected toward cabotegravir.

A substantial proportion of PLWH had resistance to NNRTIs and NRTIs. Presumably, the presence of NNRTI mutations reflects decades of prior NNRTI use for ART and perinatal transmission prevention in Botswana (although none were identified in the ART-naïve individuals). Botswana has a well-established ART program that began in 2002, initially utilizing NNRTI- and NRTI-based regimens until the adoption of dolutegravir as the preferred first- and second-line therapy in 2016. Despite the reduced use of NNRTI- and NRTI-based ART in recent years, resistance to these drug classes remains prevalent due to cross-resistance within ARV classes.^29^ Resistant HIV strains that developed during NNRTI or NRTI use can become archived in the reservoirs.^29,30^ Even after switching to dolutegravir, these resistant strains can reactivate and contribute to ongoing viral replication if the current ART does not fully suppress them. HIV strains carrying some mutations including M184V and K65R may persist because these mutations do not significantly impair the virus’s replication capacity.^31–33^ The persistence of NNRTI- and NRTI-associated resistance from earlier regimens can significantly impact treatment outcomes, resistance management and the effectiveness of new ART strategies. This emphasizes the importance of routine resistance testing and a consideration of prior treatment history when designing ART options.

Some studies have previously indicated that the presence of NNRTI-associated resistance mutations can reduce the effectiveness of dolutegravir.^9,10,34^ A study in Nigeria suggested that post-exposure resistance to NRTI-based ART may increase the risk of development of dolutegravir-associated resistance. Among 33 participants who were failing dolutegravir-based ART, 52% (17) and 73% (24) had NRTI- and NNRTI-associated resistance.^35^ In our cohort, among PLWH with INSTI-associated resistance mutations, two had dolutegravir-associated resistance and one amongst the two had NRTI- and NNRTI-associated resistance mutations, which cannot be used to conclude the link of NNRTI and NRTI with the emergence of dolutegravir-associated resistance due to low sample size. The NADIA trial reported no evidence of the link of NRTI-associated resistance with the development of dolutegravir-associated resistance although the assessment was done in virologically failing with VL ≥ 1000 copies/mL only.^16^ Some studies have assessed PLWH on dolutegravir like our study. However, they reported evidence on the link of NRTI-associated resistance with increased risk for the emergence of dolutegravir-associated resistance.^36,37^ More studies are warranted to intensively assess how pre-existing resistance towards NNRTI and NRTI influences the virological and resistance outcomes of dolutegravir in both ART-naïve and ART-experienced populations.

Our study findings indicate that HIV DRMs are a concern among both individuals with LLV and those who have VF. This finding is consistent with a study done in China that has reported a high prevalence of DRMs in PLWH with LLV^38^ and also a case of high-level resistance towards dolutegravir at LLV.^39^ PLWH with dolutegravir-associated resistance may not experience virologic failure immediately due to the fitness of mutations present and therefore may experience incomplete viral suppression or low-level viraemia. This finding highlights the need for continual HIV drug resistance monitoring among individuals with LLV in the era of dolutegravir-based ART.

The study had several limitations. This study had a relatively small sample size due to the high viral suppression rate in Botswana. Similarly, we used residual samples from the northern part of the country in the nation’s second-largest city, leaving out other highly populated areas with relatively high HIV prevalence. A key limitation of the study is low genotyping success, partly due to about 30% of the samples being from individuals with LLV, where genotyping failure rates were high. Since these samples were initially collected for VL testing, handling factors may have affected sample quality and further reducing the genotyping success. This reduction in sample numbers may have limited statistical power and potentially led to an overestimation of HIV DRMs. Despite these limitations, the study provides valuable insights into HIV DRMs in PLWH on dolutegravir-based ART with detectable VL, emphasizing the influence of previous ART regimens. Some individuals lacked historical ART information, which prevented the study from distinguishing between those who were switched from NNRTI-based ART to dolutegravir-based ART due to updated HIV treatment guidelines in Botswana either with previous VF or viral suppression and understanding how previous exposure to other ARTs before dolutegravir influenced the development of DRMs. One of the study limitations is the unavailability of the resistance data at earlier timepoints to assess the evolution of the HIV DRMs. Despite these limitations, the study successfully detected DRMs associated with NRTIs, NNRTIs, PIs and INSTIs among PLWH on ART with a detectable VL > 200 copies/mL, using VL residual samples from those on dolutegravir-based ART. The possibility of using confirmed detectable VL residual samples for HIV drug resistance testing will shorten the turnaround time, especially in resource-limited settings where VL results turnaround time is lengthy. This will eliminate the process of requesting a specimen for HIV drug resistance testing.

### Conclusion

Among PLWH on ART with viraemia in the Botswana National ART program, a third of individuals had DRMs, with similar prevalence among those with LLV or VF. There was a low prevalence of dolutegravir-associated resistance mutations, but a high prevalence of resistance to NRTIs and to NNRTIs (despite little current NNRTI use in the country). This may warrant a reconsideration of the importance of LLV in national drug ART policies.

## Supplementary Material

dkae472_Supplementary_Data

## Data Availability

All relevant data are within the paper, including the figures and tables. All 58 HIV protease, reverse transcriptase and integrase generated sequences are submitted to National Center for Biotechnology Information (NCBI) GenBank and awaiting the accession numbers.

## Supplementary data

Table S1 is available as Supplementary data at *JAC* Online.
