# Supplementary material for: High prevalence of reverse transcriptase inhibitors associated resistance mutations among people living with HIV on dolutegravir-based antiretroviral therapy in Francistown, Botswana
Source: J Antimicrob Chemother. 2025 Jan 7;80(3):767–76. doi: 10.1093/jac/dkae472 (PMC11879200; doi:10.1093/jac/dkae472)
Supplement: dkae472_Supplementary_Data [file dkae472_supplementary_data.docx]

Supplementary table 1. Amplification and genotyping outcomes by viral load groups

|  | Amplification success | | Sequencing Success** | |
| --- | --- | --- | --- | --- |
|  | HIV PRRT | HIV IN | HIV PRRT | HIV IN |
| Total (n=100) | 68 (68.0%) | 71 (71.0%) | 49/68 (72.1%) | 51/71 (71.8%) |
| LLV (200-999 copies/mL) (n=30) | 16 (53.3%) | 21 (70.0%) | 9/16 (56.3%) | 11/21 (52.4%) |
| VL≥1000 copies/mL (n=70) | 51 (72.9%) | 52 (74.3%) | 40/51 (78.4%) | 40/52 (76.9%) |

*HIV IN-HIV integrase region; HIV PRRT- HIV protease and reverse transcriptase region; n-total number of samples; ** Sequencing success was calculated using the number of successfully amplified samples as the denominator.*
